# Outliers and anomalies in training and testing datasets for AI-powered morphometry—evidence from CT scans of the spleen

**DOI:** 10.3389/frai.2025.1607348

**Published:** 2025-07-15

**Authors:** Yuriy Vasilev, Anastasia Pamova, Tatiana Bobrovskaya, Anton Vladzimirskyy, Olga Omelyanskaya, Elena Astapenko, Artem Kruchinkin, Novik Vladimir, Kirill Arzamasov

**Affiliations:** ^1^Research and Practical Clinical Center for Diagnostics and Telemedicine Technologies of the Moscow Health Care Department, Moscow, Russia; ^2^National Medical and Surgical Center named after N.I. Pirogov of the Ministry of Health of the Russian Federation, Moscow, Russia; ^3^I.M. Sechenov First Moscow State Medical University of the Ministry of Health of the Russian Federation (Sechenov University), Moscow, Russia; ^4^Moscow Technical University - MIREA, Ministry of Science and Higher Education, Moscow, Russia

**Keywords:** outliers, anomalies, dataset, machine learning, statistics, spleen, computer tomography

## Abstract

**Introduction:**

Creating training and testing datasets for machine learning algorithms to measure linear dimensions of organs is a tedious task. There are no universally accepted methods for evaluating outliers or anomalies in such datasets. This can cause errors in machine learning and compromise the quality of end products. The goal of this study is to identify optimal methods for detecting organ anomalies and outliers in medical datasets designed to train and test neural networks in morphometrics.

**Methods:**

A dataset was created containing linear measurements of the spleen obtained from CT scans. Labelling was performed by three radiologists. The total number of studies included in the sample was *N* = 197 patients. Using visual methods (1.5 interquartile range; heat map; boxplot; histogram; scatter plot), machine learning algorithms (Isolation forest; Density-Based Spatial Clustering of Applications with Noise; K-nearest neighbors algorithm; Local outlier factor; One-class support vector machines; EllipticEnvelope; Autoencoders), and mathematical statistics (z-score, Grubb’s test; Rosner’s test).

**Results:**

We identified measurement errors, input errors, abnormal size values and non-standard shapes of the organ (sickle-shaped, round, triangular, additional lobules). The most effective methods included visual techniques (including boxplots and histograms) and machine learning algorithms such is OSVM, KNN and autoencoders. A total of 32 outlier anomalies were found.

**Discussion:**

Curation of complex morphometric datasets must involve thorough mathematical and clinical analyses. Relying solely on mathematical statistics or machine learning methods appears inadequate.

## 1 Introduction

The advancement of artificial intelligence (AI) technologies for enhancement of priority sectors such as healthcare is a key component of the national agenda in many countries (Decree of the President of the Russian Federation, 2019; Mashraqi and Allehyani, 2022; Vasilev et al., 2024) (Supplementary Figure S1).

Dataset curation is an essential component of digitalization. Datasets are critical for both machine learning algorithms and AI model testing. One of the key factors is the quality of validation and test datasets. The dataset evaluation methods, labelers’ quantity and training, and quality of data (i.e., images, tables, and annotations)—are critical (Vasilev et al., 2024). This study assesses and identifies outliers and anomalies that emerge during curation of training and testing datasets for machine learning applications in computer vision, specifically for automating measurements in diagnostic imaging (regression labelling) and morphometrics.

Since 2020, a large-scale experiment has been conducted in Moscow, Russia, to explore innovative computer vision technologies for medical image analysis and enhancement of the healthcare system (hereinafter - the Experiment). Over this period, the municipal public healthcare system was enhanced with AI models. Since the beginning, the models have evolved and now demonstrate high efficiency in detection and classification tasks (Mosmed.AI, 2025).^1^ This is evidenced by the fact that the second reading of screening mammograms in Moscow is conducted by an AI model rather than a radiologist (Vasilev et al., 2023).

The next task for the developers was to create AI models that automate routine measurements (morphometry). This required generation of complex datasets with measured anatomical structures. Routine data from radiological studies exhibit several characteristics: ambiguity, lack of standardization, large per-patient data volumes, dynamic parameters, and abundance of techniques for organ measurements (Vasilev et al., 2023). Consequently, when curating datasets for training and testing the morphometric AI, we encountered a lack of uniform approaches or clear roadmaps. Certain challenges were associated with detecting outliers and anomalies during regression labelling of organs (linear dimensions, angles between organ structures, volumes, indices, and areas).

This paper defines an outlier as an observation (measured linear dimension of an organ) that significantly deviates from other observations, suggesting an error. This may result from physician input errors or measurement errors due to varying approaches to determining organ linear dimensions, influenced by radiologist expertise. Outliers in the final dataset can significantly impact machine learning algorithm training. Since even isolated values can substantially influence machine learning algorithms, neglecting dataset standardization could compromise the AI model, or lead to incorrect assessment and misinterpretation of radiological findings (Wada, 2020; Makarov and Namiot, 2023).

The goal of outlier identification goes beyond their immediate removal from dataset. Outliers can be categorized as either errors, requiring revision, correction, or removal, or anomalies (hereafter - *anomalies*) (Wada, 2020; Yepmo et al., 2022; Foorthuis, 2021). *Anomalies* are observations that deviate from other measurements but are not caused by input or technical errors. They are of particular interest, necessitating careful analysis to determine their cause, such as a rare but possible organ size. Unreasonable removal of such data can compromise representativeness and, consequently, significant interpretation errors (Yepmo et al., 2022; Gaspar et al., 2011). Furthermore, abnormal organ size values can be of particular interest to researchers.

Consequently, the analysis of *outliers* and *anomalies* includes the following tasks:

1. Identifying observed values as outliers;
2. Reviewing data acquisition procedures and understanding the cause of outliers;
3. Identifying abnormal values, as they may be of particular research interest, and considering them separately.

Although outlier identification has been addressed in numerous scientific papers since the mid-19th century (Peirce, 1852), it remains highly relevant (Wada, 2020; Yepmo et al., 2022; Foorthuis, 2021). Currently, outlier and anomaly identification utilizes various methods, including machine learning (Makarov and Namiot, 2023; Nassreddine et al., 2023; Diers and Pigorsch, 2022), in addition to mathematical statistics (Sidnyaev and Battulga, 2024; Sysoev and Scheglevatych, 2019).

Development of datasets for morphometric AI models required comparison between mathematical statistics and machine learning approaches using a dataset of spleen linear measurements. Three radiologists labelled the dataset. We considered the causes of outliers and anomalies, and proposed options for their occurrence and prevention. Thus, this paper seeks to advance the enhancement of healthcare AI, particularly in creating reliable systems that automate radiological measurements. This work addresses identification and processing of outliers and anomalies in morphometric datasets. Furthermore, it aligns with current global AI development strategies and modern trends in AI implementation in “digital healthcare.”

The goal of this paper is to identify effective methods for detecting and processing outliers and anomalies in radiological morphometric datasets, particularly for regression labelling of organ linear dimensions in computed tomography.

## 2 Materials and methods

The data were acquired during the Experiment (ClinicalTrials.gov identification code—NCT04489992).

### 2.1 Dataset

#### 2.1.1 Population data

The dataset comprises computed tomography (CT) images of abdominal organs that includes linear dimension measurements of the spleen. The data acquired in Moscow medical facilities between April 1, 2023, and May 28, 2024, were extracted from the Unified Radiological Information Service of the Unified Medical Information and Analytical System of Moscow (ERIS EMIAS). The data were anonymized using dedicated anonymization software (Vasilev et al., 2025). The dataset includes: 197 patients (89 men, 108 women). Age: minimum 18 years, maximum 99 years, median 61 years.

To prepare the dataset for evaluating AI models in spleen morphometry, sample size was calculated using Scipy and NumPy libraries in Jupyter Notebook. The null hypothesis (H0) suggested that the AI model would yield correct measurements for at least 81% of the studies. The anticipated AI performance was at 86%. With statistical power of 80% and one-sided significance of 0.025, a sample size of 379 measurements was determined. This was rounded up to 400 to account for potential data rejection. Three defective studies were excluded (low image quality, absence of the spleen), resulting in 394 measurements from 197 studies.

CT acquisition parameters: native study, slice thickness ≤1.5 mm, windowing “soft tissue (standard) kernel.”

*Inclusion criteria:* patient age over 18 years.

*Exclusion criteria:* artifacts, positioning defects, low image quality, incorrect slice thickness, oral contrast, and spleen absence.

#### 2.1.2 Dataset labelling

Three radiologists independently labelled the studies. Requirements to labelers: radiologist certification and at least 3 years of abdominal CT experience.

Spleen diameter (largest anterior–posterior axial measurement) and thickness (the largest perpendicular dimension to the diameter in the axial plane) (Gaillard et al., 2009) were measured using a DICOM viewer (AGFA, Belgium), rounded to the nearest whole number, and recorded in an Excel table (.xlsx).

#### 2.1.3 Units of measurement

The Russian Federation uses the International System of Units. All spleen measurements were in millimetres (mm), ranging from 1 to 100 mm.

### 2.2 Data analysis

Statistical parameter calculation, machine learning, and data visualization were performed in Python (version 3.11.5) using the following libraries: *numpy*, *pandas*, *matplotlib*, *seaborn*, *scipy*, and *sklearn*, in their latest versions as of May 1, 2024.

Two method groups were used: mathematical statistics and classical machine learning, with a Kolmogorov–Smirnov test for normal distribution.

A selective literature review was conducted using various databases (PubMed, ScienceDirect, Google Scholar, Scopus, elibrary, etc.), though it was not the primary focus of this paper. Several mathematical statistics and machine learning method groups commonly used for outlier and anomaly detection in medical datasets were identified. Further spleen dataset analysis was performed using these methods.

#### 2.2.1 Mathematical statistics and visual methods

Interquartile range (1.5 IQR) (Vinutha et al., 2018): a measure of dispersion reflecting the data spread. Specifically, it is defined as the difference between the upper (Q3) and lower (Q1) data quartiles (per scipy library documentation).

*Z-score:* a method based on data standardization (Carey and Delaney, 2010), requiring value recalculation using the formula (1):


(1) $$Z_{i}=\frac{x_{i}-\mu }{\sigma }\;$$

where x is the measurement result, *μ* is the mean value, and *σ* is the standard deviation. A Z-score exceeding three SD from the mean is considered an outlier.

*Grubbs’ test:* used (Adikaram et al., 2015) for samples with more than six observations (*n* > 6) to identify whether the largest or smallest value is an outlier. It detects isolated outliers (maximum or minimum), requiring iterative application. The null and alternative hypotheses are as follows (statistical significance *α* = 0.05):

*H0:* The largest (smallest) value is not an outlier.

*H1:* The largest (smallest) value is an outlier.

Suitable for isolated outlier detection; for multiple outliers, other methods are preferred. More suitable for outlier detection in normally distributed data (Davies and Gather, 1993).

*Rosner’s test* (Rosner, 1975; Rosner, 1983): used for samples with more than 20 observations (*n* > 20) to detect multiple outliers simultaneously. The method assumes a normal data distribution. The method compares each observation with other values. Although this method can detect outliers, we did not consider it as it is not applicable to our data.

#### 2.2.2 Machine learning

*Isolation forest*: a robust outlier detection algorithm (resistant to small data fluctuations). The algorithm relies on decision tree principles and the ensemble random forest method (Sysoev and Scheglevatych, 2019; Popova, 2020). The algorithm randomly selects a feature and a split within that feature’s range. Observations less than or equal to the split go to the left child node; those greater go to the right. This process is repeated recursively across the dataset.

The following algorithm settings were used: contamination = 0.05, random_state = 3,000. Principal component analysis was used for outlier visualization, though it does not clearly identify outlier values. Therefore, a table of potential outlier values was generated for comprehensive analysis. Principal component analysis (Greenacre et al., 2022) reduced data dimensionality to three principal components (considered necessary and sufficient), enabling three-dimensional data visualization. Where there are multiple features, the method allows identifying the total number of potential outliers. This technique is useful for visualizing outlier distribution across features.

*Density-based spatial clustering of applications with noise (DBSCAN):* the algorithm (Monalisa and Kurnia, 2019; Schubert et al., 2017) identifies clusters in data and outliers, regardless of cluster shape. It identifies high-density feature kernels and expands clusters with them. Suitable for data with clusters of similar density. Requires careful manual parameter selection, which can be challenging for multidimensional data. DBSCAN defines clusters based on two parameters: Eps (maximum distance between two points to be considered neighbours) and min_samples (minimum number of neighboring points to qualify as a core point). If the *ε*-neighborhood has fewer than min_samples points, the point is not a core and may be considered noise.

In this study: spleen diameter - ε (eps) = 30, min_samples = 20; spleen thickness - eps = 9, min_samples = 15. Parameters were manually adjusted.

*K-nearest neighbors algorithm (KNN):* this supervised learning method (Monalisa and Kurnia, 2019) was used in a non-conventional way. The dataset lacked predefined “outlier” or “non-outlier” labels, as it was not possible to know in advance whether it contained outliers or anomalies. Therefore, the method relied entirely on threshold values. The threshold values for outlier identification were set manually. After model training, the kneighbors method identified the k nearest neighbors for each observation, and distances to these neighbors were calculated. If k is too small, there is a risk of missing an outlier cluster; if k is too large, regular points may be misclassified as outliers (Wang et al., 2021). In this study, n_neighbors = 3.

*Local outlier factor (LOF):* this method (Popova, 2020; Xu et al., 2022; Dulesov and Bayshev, 2023) uses data point density to detect outliers. Like KNN, LOF uses k-nearest neighbor distance estimation for outlier detection. It calculates the LOF metric based on sample local density and its k-nearest neighbors. This method is useful when the outlier status depends on the neighborhood of the data point, not the entire dataset. The method assigns each observation an outlier rate based on its isolation compared to neighboring observations (data points).

The following parameters were used: n_neighbors = 10, contamination = 0.5, novelty = False.

*One-class support vector machines (OSVM):* a popular outlier detection method (Ji and Xing, 2017), but sensitive to noise as it treats all observations equally. It is more suitable when all observations in the dataset follow a normal distribution. Since this is a supervised learning method, the dataset must be split into training and validation sets. The method constructs a nonlinear surface around the origin. A cut-off threshold (*gamma*) (Wang et al., 2018) can be set for anomalous data, useful for non-normally distributed datasets. The *nu* parameter controls the outlier proportion.

The following parameters were used: kernel = “rbf,” gamma = 0.001, nu = 0.03.

*EllipticEnvelope:* an outlier detection method (Kim et al., 2019) effective for normally distributed or time-series datasets.

The following settings were used: contamination = 0.03, random_state = 0.

*Autoencoders:* a method based on autoencoder, which is a neural network, was used to detect anomalies (Abhaya and Patra, 2023). It is trained to recover the input data from its compressed latent representation. The autoencoder architecture included 6 input features (spleen diameter and thickness along three dimensions), an encoder with layers of dimensionality 8, 4, 2 and a similar decoder, with LeakyReLu activation function (negative_slope = 1) and parameter optimization using the Adam algorithm with a learning step of 0.001. StandardScaler was used to normalize the data. Training was performed for 400 epochs using the MSE (mean square error) loss function. Outliers were defined as points whose reconstruction errors exceeded a threshold set at the 90th percentile level.

#### 2.2.3 Data visualization

Outlier or anomaly presence can be determined by using methods other than mathematical statistics or machine learning. Visual statistical methods can be used independently of algebraic calculation methods.

*Boxplot:* box and whisker plots (Sim et al., 2005) are commonly used to present dataset details. It allows preliminary exploratory data analysis and identify outliers or extreme values (anomalies). This method provides insight into location, distribution, and asymmetry of data points. However, the method has limitations, especially with non-parametric data distributions.

*Histogram:* despite its seeming simplicity and applicability restrictions (Goldstein and Dengel, 2012), this method offers speed, accuracy, and adaptability to various data distributions. Outlier detection is highly dependent on histogram bin diameter and data accumulation per bin. Therefore, this method, like the boxplot, is effective for initial exploratory data analysis. The following parameters were used: bins = 30.

*Heat map:* this graphical method is used only in combination with other methods (DeBoer, 2015). In our case, it is associated with the Z-score. No specific parameters were used for the heat map.

*Scatter plot*: scatter plots can help detect outliers in visualized data clusters (Yuan and Hayashi, 2010). It can be used to identify and explain the behaviour of observed data points. No special settings were used for this method in our study. Interpretation of results.

An expert radiologist (over 5 years of CT experience) reviewed values identified as outliers or anomalies and interpreted the corresponding studies.

### 2.3 Validation of the use of methods in AI testing

To illustrate the use of our outlier search methods, we studied their impact on the testing process of an open source algorithm for spleen segmentation - medical open network for artificial intelligence (MONAI) (Project MONAI, 2025). For testing we used our dataset, where outliers and anomalies in the data had already been found previously, using the above methods.

Using MONAI, we obtained spleen segmentation masks. Additionally, using the MONAI algorithm (after some refinement), we calculated the diameter and thickness of the spleen. The obtained dimensions were compared with data from three radiologist. We evaluated the hit in the range (minimum and maximum value from 3 doctors) of the measurements performed by the AI. We calculated the percentage of hits in the range. Then the expert reviewed those cases where there were input and measurement errors.

## 3 Results

Exploratory analysis and descriptive statistics calculation are mandatory data analysis steps, allowing researchers to formulate preliminary hypotheses that are subsequently tested. The results of basic descriptive statistics calculations for spleen thickness and diameter measurements by three radiologists are presented in Supplementary Table S1.

### 3.1 Data obtained using visualization methods

**Figure 1:**
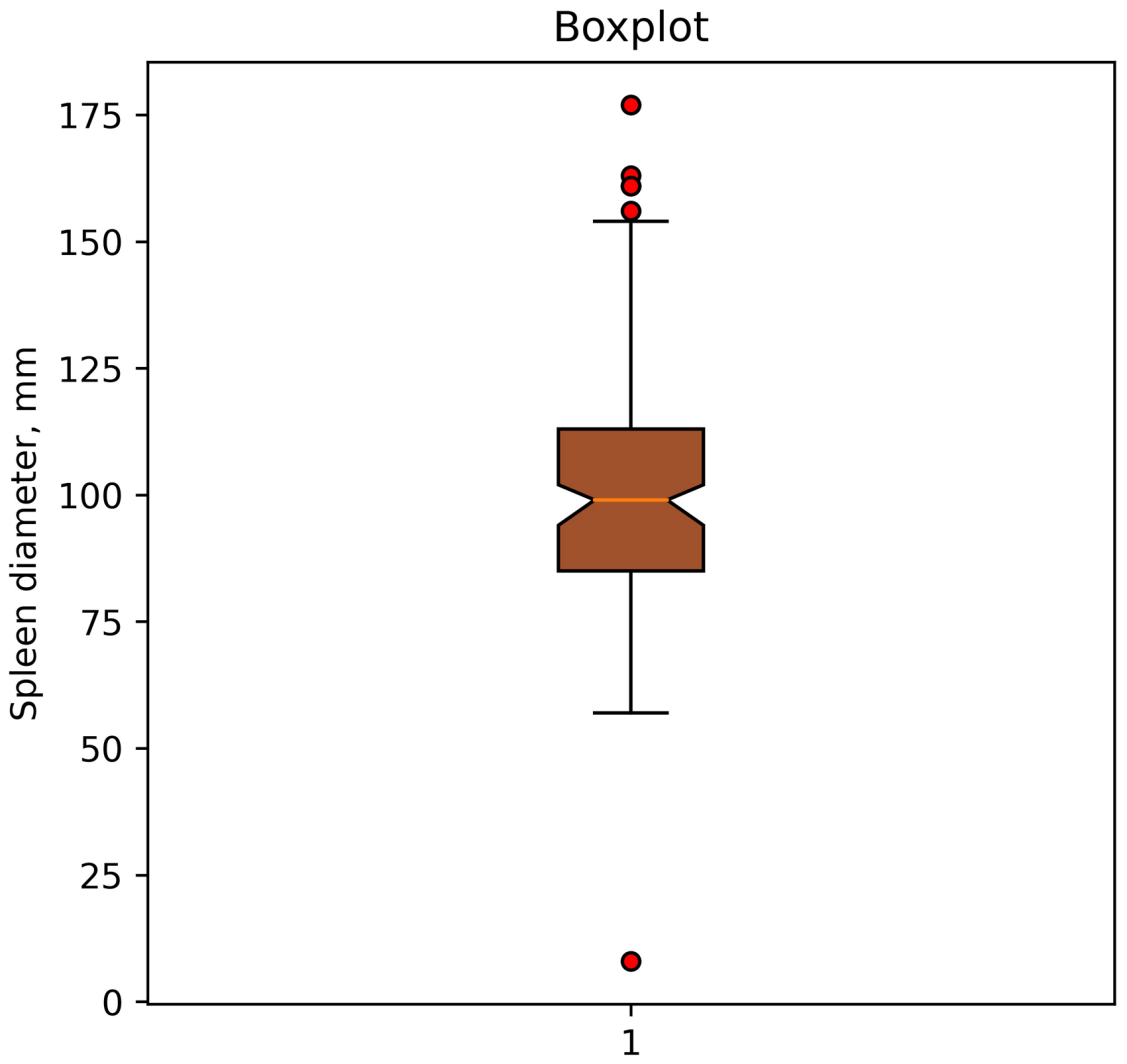
Example of outlier identification—boxplot. Dots indicate potential outliers or anomalies in spleen diameter data (mm).

1. Finding outliers using a boxplot. Shown in Figure 1. Potential outliers are values outside the boundaries of (Q3, Q1) ± 1.5 IQR.

In this case, the method classified spleen diameter measurements >150 mm and <50 mm as outliers.

**Figure 2:**
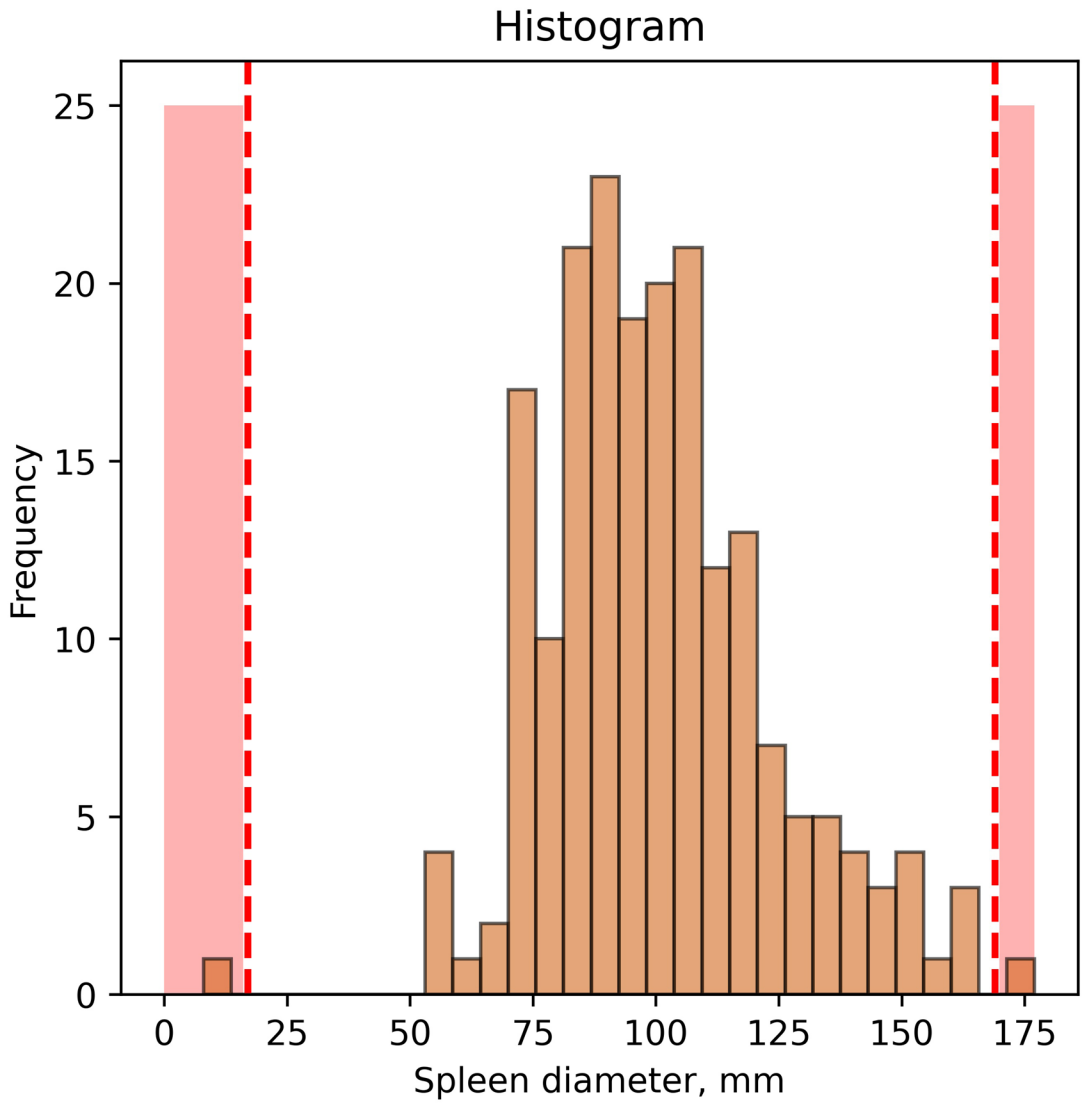
Example of outlier identification—histogram. Red dotted lines and red colouring indicate potential outliers/anomalies.

2. An example of outliers found using a histogram is shown in Figure 2.

In this case, interpretation of visual data depends entirely on researcher opinion. In our study, spleen diameter of 175 mm or more and below 50 mm were considered outliers.

**Figure 3:**
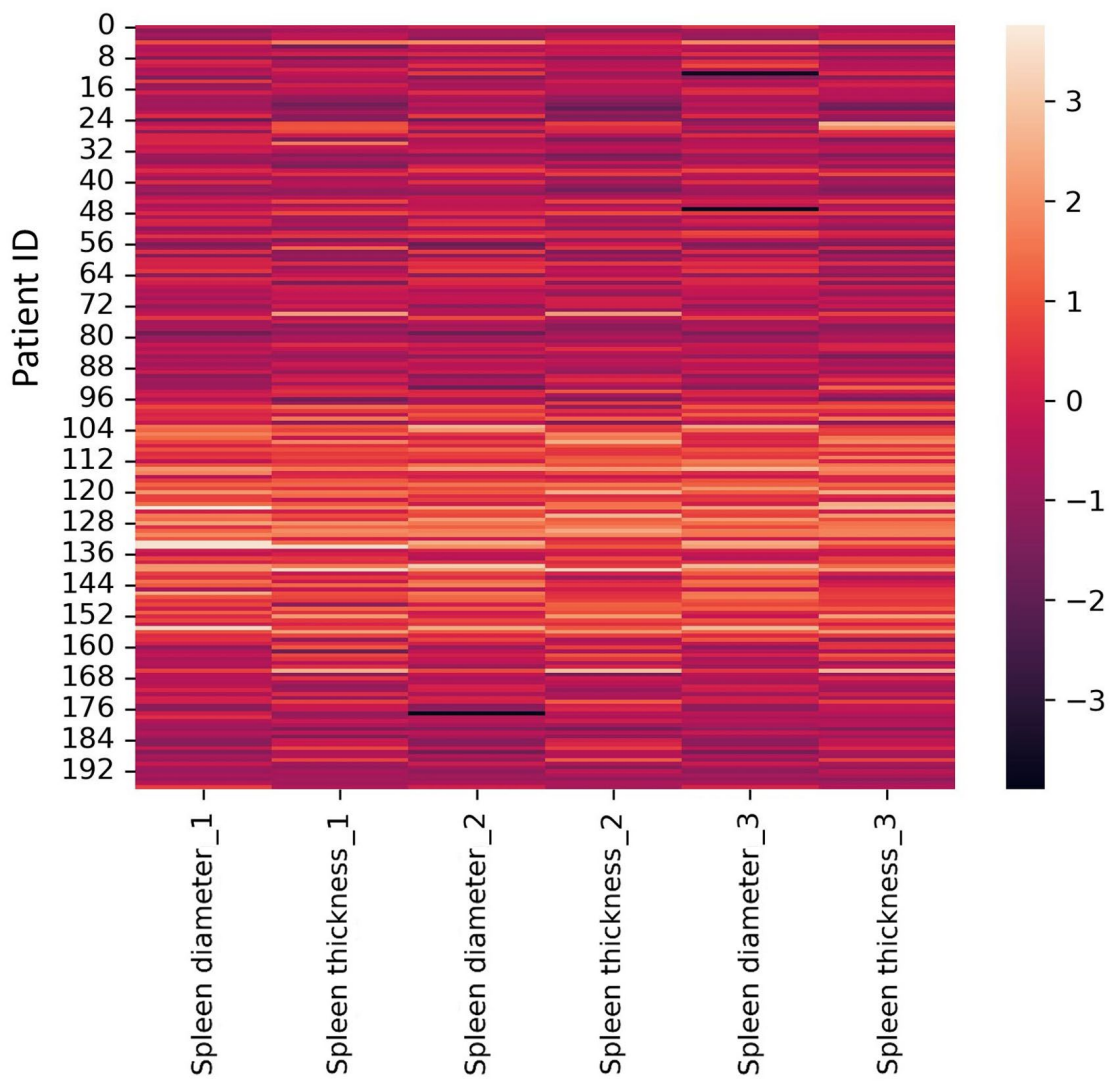
Heat map constructed for Z-scores (±3σ). Diameter and thickness units are millimeters (mm).

3. A heat map constructed using Z-scores: this method normalizes all dataset values. We used a heat map for visualization. A heat map is a graphical data representation where dataset values are represented by matrix colors. This method allows to visually assess features whose values differ significantly from the average, indicating potential outliers. The resulting heat map is shown in Figure 3.

The heat map interpretation: black bars are outliers, lighter areas are common measurements among radiologists in our dataset. Observed outliers: Radiologist #3–8 and 48 mm (spleen diameter); Radiologist #2–176 mm (spleen diameter). A limitation of this method is the difficulty in accurately identifying outliers and their exact values in large datasets. The most common spleen diameter measurement range is 96 mm – 160 mm.

**Figure 4:**
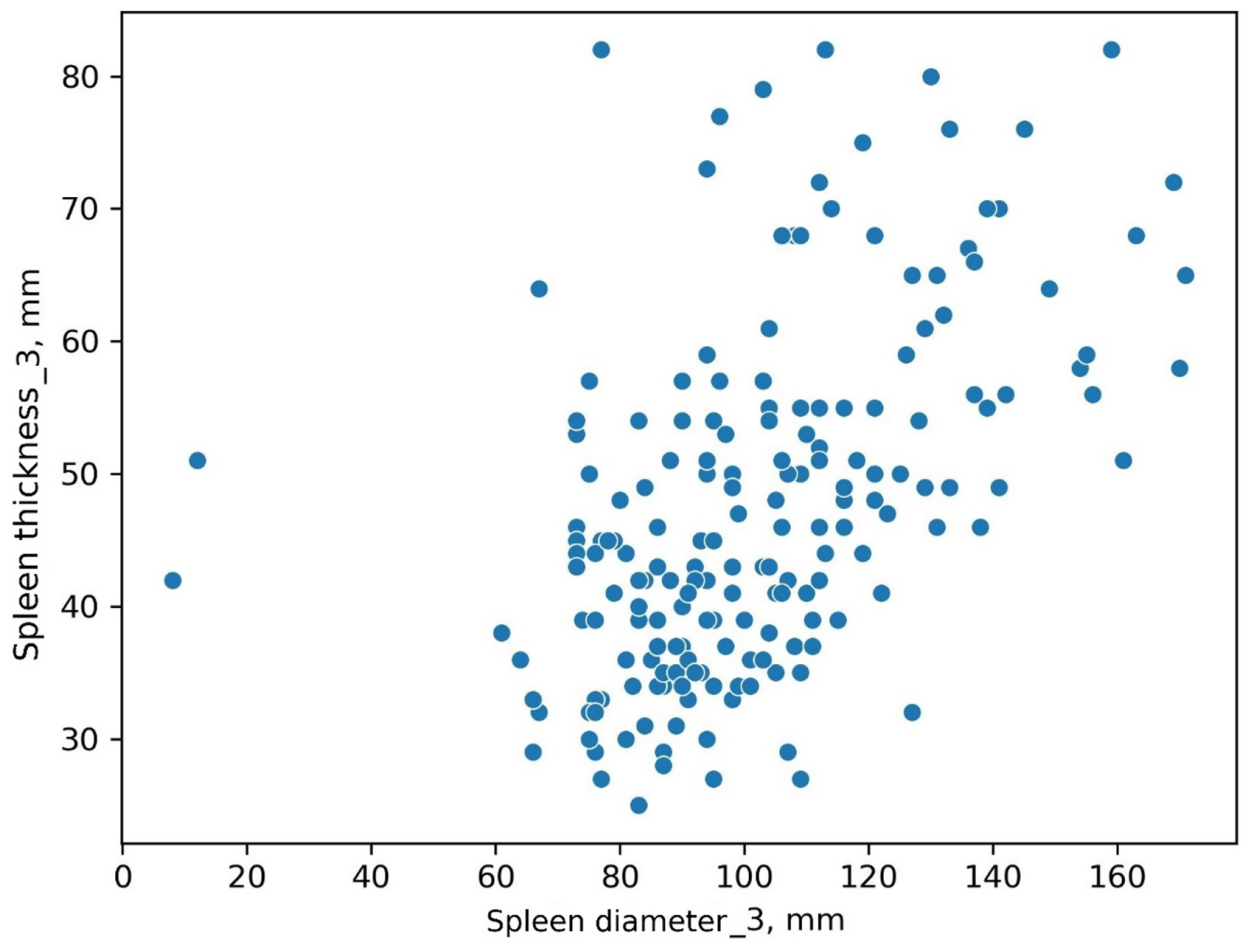
Scatter plot. Spleen diameter and thickness, Radiologist #3.

The scatter plot was constructed using both spleen diameter and thickness values. Figure 4 shows two values that differ from the others.

It is difficult to determine if these are input error outliers or two patients with unusually small spleens. This graphical method allowed us to define a task: review this radiologist’s results for these two patients, clustered separately.

### 3.2 Outliers detected by mathematical statistics methods

Table 1 summarizes the number of outliers (or anomalies) detected by mathematical statistics and graphical methods.

**Table 1:** Number of outliers identified by mathematical statistics methods for spleen thickness and diameter measurements across radiologists.

Method	Thickness 1	Thickness 2	Thickness 3	Diameter 1	Diameter 2	Diameter 3
Hist (bins = 197)	2	4	0	5	2	2
Boxplot	2	4	0	6	5	8
Z-score	0	0	1	4	1	3
Grubbs test	0	0	0	0	2	4
Rosner test	0	0	0	4	1	2

The diameter and thickness indices indicate the radiologist who conducted the study.

The two-sided Grubbs test shows Radiologist #1’s maximum spleen diameter values (Supplementary Table S1) are not outliers. Radiologist #2’s maximum and minimum spleen diameter measurements are outliers. Radiologist #3 has four outliers: two maximum and two minimum values. Grubbs and Rosner tests assume normally distributed data, which our data did not follow, so these results were excluded from analysis.

### 3.3 Outlier evaluation using machine learning

Machine learning methods detected outlier or anomaly signs in 24 patients.

**Figure 5:**
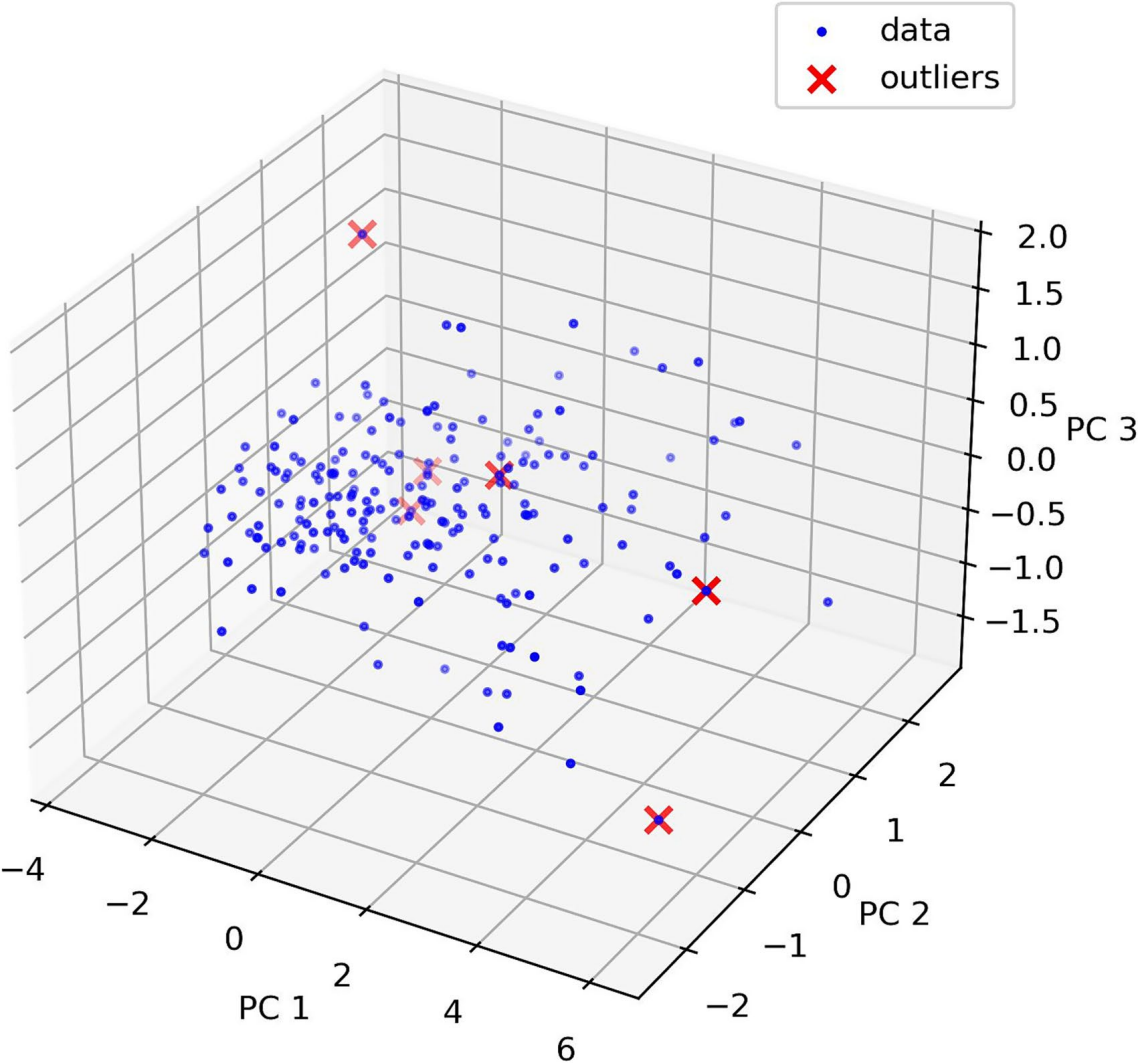
Outliers found using isolation forest and principal component analysis. PC is the component number (three main components). PC1 explains the largest data variance, indicating maximum data variation direction. PC2 explains the second largest data variance. PC2 is orthogonal to PC1. PC3 is the third axis orthogonal to PC1 and PC2.

To demonstrate the Isolation Forest method, a graph (Figure 5) was constructed showing outliers using principal component analysis. Principal component analysis creates new linearly independent variables by combining original variables. Principal component (PC) is the coordinate axis that maximizes data variance.

Principal component analysis interpretation is difficult due to data dimensionality reduction that makes perception inconvenient. This graph shows data location in reduced-dimensionality space, clarifying why some data points were considered outliers. Full detected outlier information is shown in Table 2. Outlier value clarification is necessary and remains at the researcher’s discretion.

**Table 2:** Suspected outliers identified using isolation forest.

Diameter 1	Diameter 2	Diameter 3	Thickness 1	Thickness 2	Thickness 3
89	115	12*	45	35	51
151	154	169*	68	72	72
151*	128	130	68	78	80
192*	156	159	71	65	82
187*	161	163	65	53	68
187*	148	156	92*	59	56
151*	177	171	68	53	65
152	153	145	91	86	76*
182*	161	170	57	59	58
115	121*	113	81	81	82

*Outliers.

**Figure 6:**
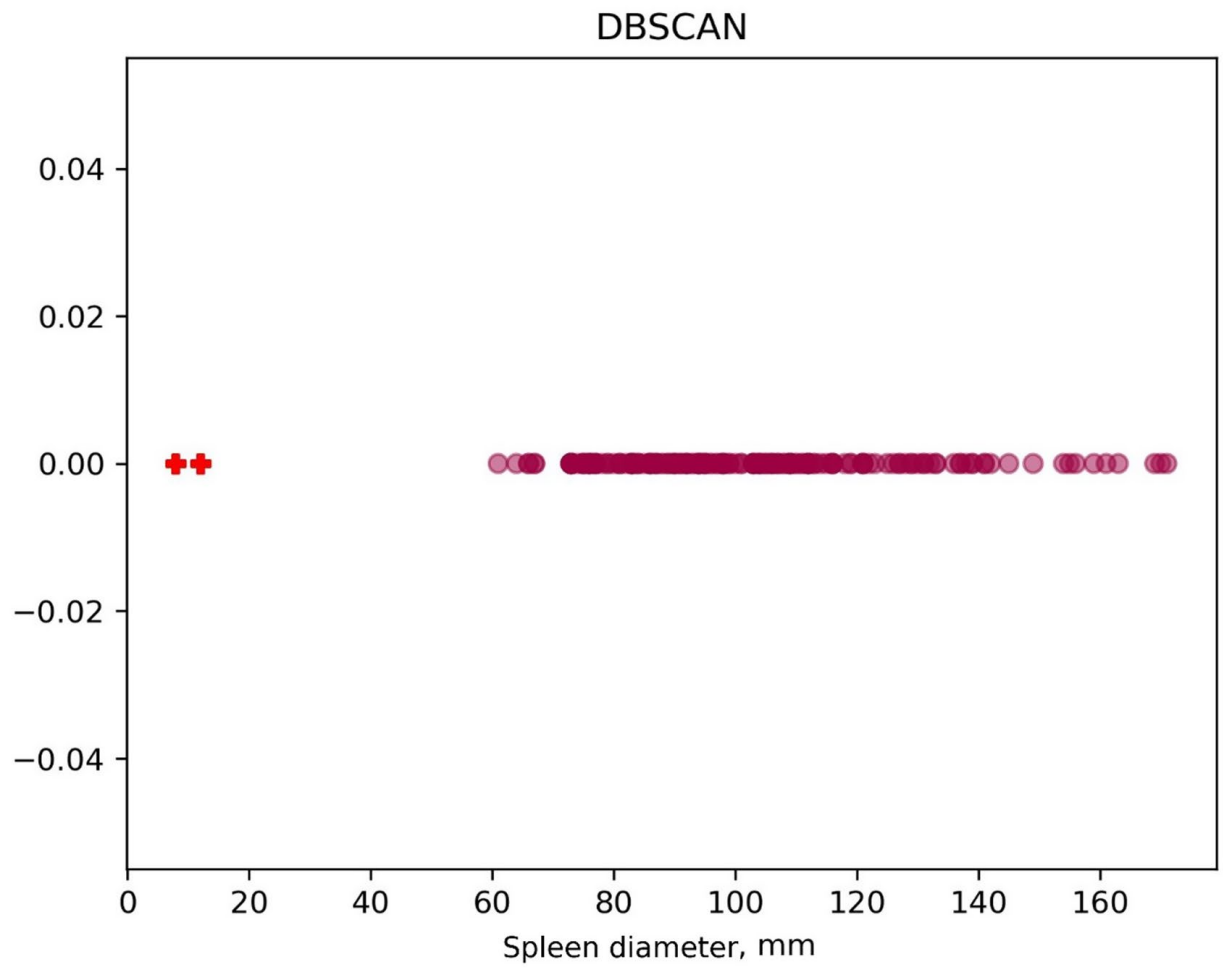
Determining outliers using DBSCAN. “+” - algorithm-found outliers.

For the DBSCAN algorithm, graphs (Figure 6) of linear value distribution were constructed. While the method makes it convenient to determine outliers, it is parameter-sensitive and outlier-insensitive. In this case, two outliers are recognized, with spleen diameter less than 20 mm.

**Figure 7:**
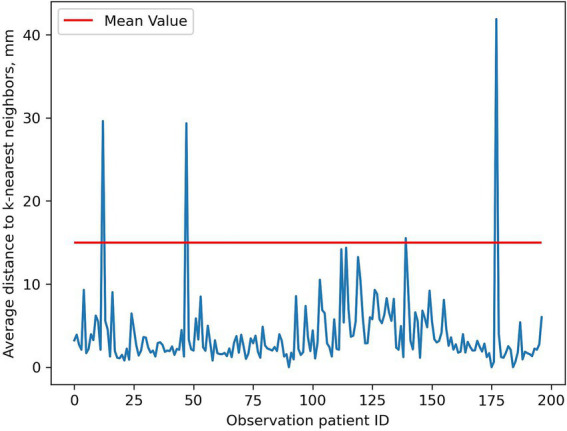
Outlier search using KNN. Spikes are potential outliers. ID, identifier.

Outliers identified with the KNN method are not entirely obvious. Visualization methods (Figure 7) simplify decision-making about presence/absence of outliers.

Result interpretation relies on the researcher. We considered all spikes above Radiologist #3’s average spleen diameter as outliers. Precise interpretation is difficult as exact outlier values are unspecified. Outlier identification requires comparison with the original table containing patient’s unique identification number (UID).

**Figure 8:**
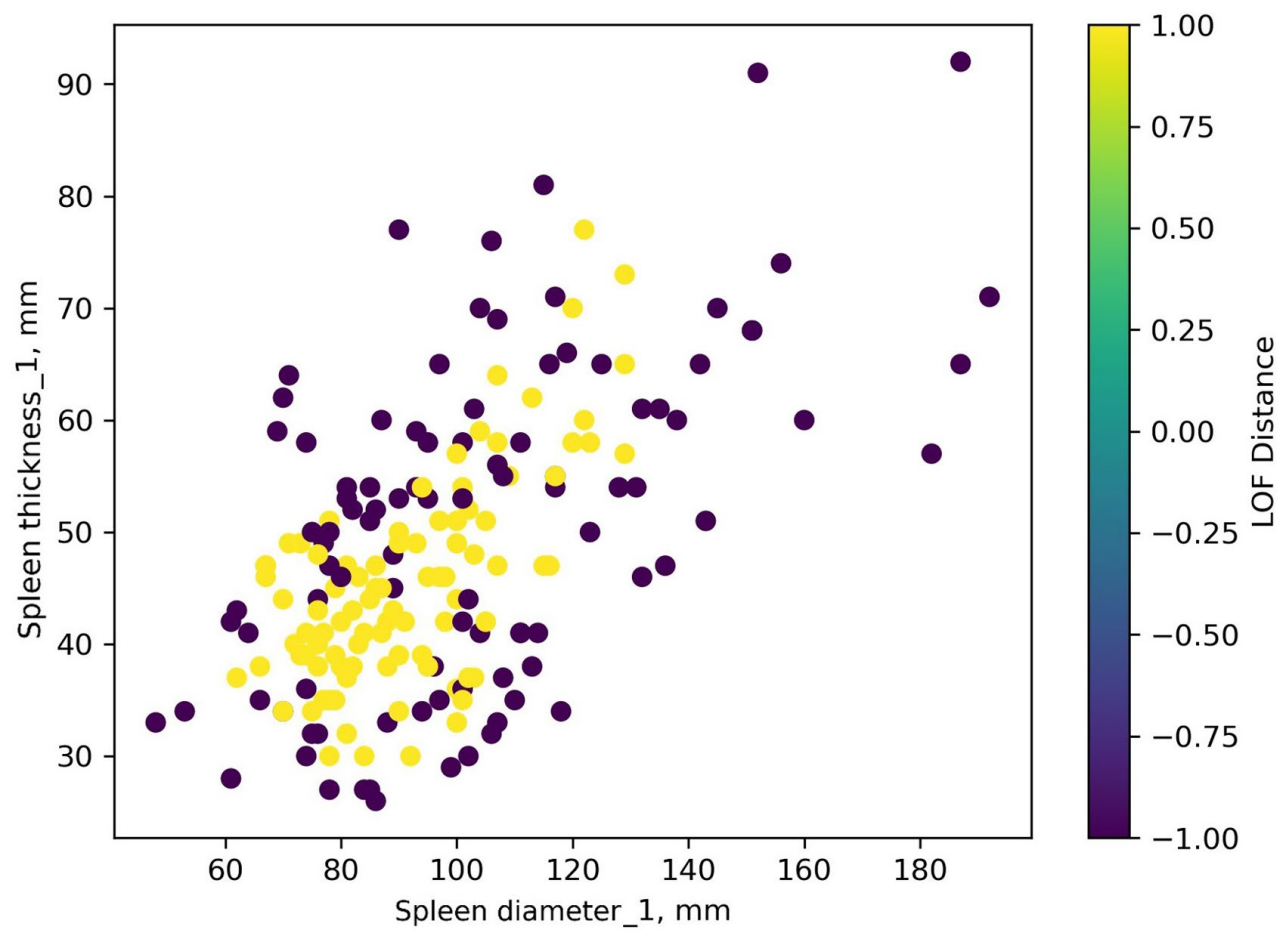
Identifying anomalies in a dataset using the LOF algorithm. Yellow points represent potential outliers.

Regardless of settings, the LOF algorithm identifies numerous dataset observations as outliers: 99 out of 197 observations were considered anomalies. An example of visualization is presented in Figure 8.

Interpreting LOF Distance values requires understanding the LOF algorithm. In our case, LOF Distance values are normalized from −1 to 1. Values close to 1 indicate an outlier. Values close to −1 indicate a point is in a dense dataset cluster. Values near 0 indicate a regular data point (there are none in this dataset, values are either outliers or a single cluster). Outlier identification threshold choice can be based on expert opinion. However, we could not find an optimal threshold (threshold set to the 95th percentile is shown in Figure 8).

**Figure 9:**
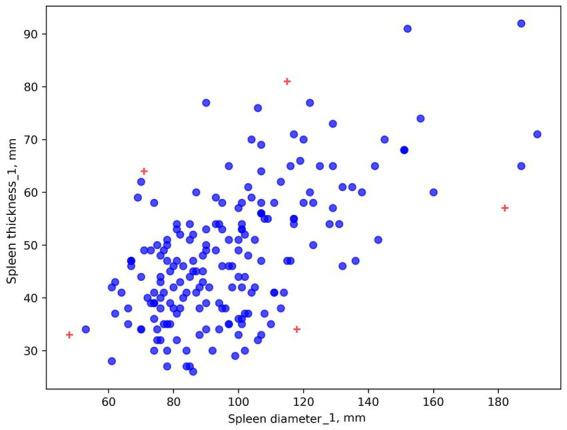
Identifying anomalies in a dataset using the OSVM algorithm. Radiologist #1’s results are shown as an example. Crosses represent potential outliers.

OSVM is also difficult to configure. It is not always clear which observation is an outlier and why. Visualization is presented in Figure 9.

Five outliers were detected in Radiologist #1’s labels. It is unclear why these specific points were considered outliers. For precise anomaly and outlier identification, an outlier table is advisable, though it may not specify spleen thickness or diameter. In this case, all red plus-marked points in Figure 9 and shown in Table 3 are considered outliers (for Radiologist #1).

**Table 3:** Outliers identified by the OSVM method in radiologist #1’s labelling.

Spleen diameter, mm	Spleen thickness, mm
48	33
71	64
118	34
182	57
115	81

Anomaly type (diameter or thickness) is indeterminate.

**Figure 10:**
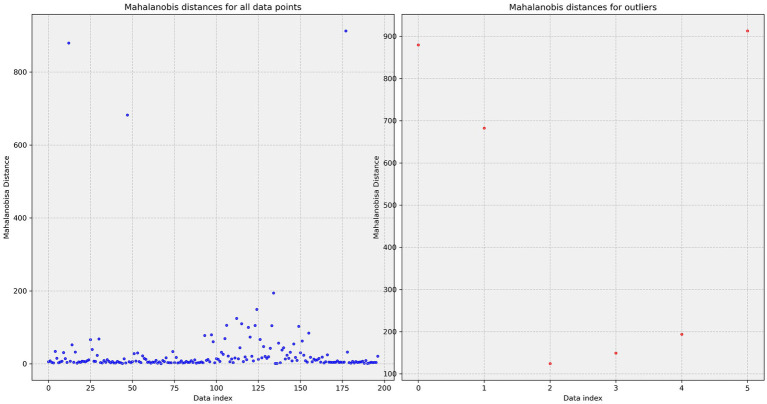
Detecting outliers using EllipticEnvelope. Calculating Mahalanobis distance.

EllipticEnvelope is convenient for anomaly or outlier detection. However, some outliers are questionable, despite the algorithm’s help with obvious outliers. Visualization is presented in Figure 10. Anomaly detection strongly depends on method settings.

An outlier table is necessary in addition to all methods. In this case, 8 mm spleen diameter is an outlier, but this is not clear from the graph. A separate table revealed six outliers: 8 mm (Radiologist #2 spleen diameter), 12 mm (Radiologist #3 spleen diameter), 8 mm (Radiologist #3 spleen diameter), 90 mm (Radiologist #1 spleen diameter), 192 mm (Radiologist #1 spleen diameter), and 92 mm (Radiologist #1 spleen diameter).

Autoencoders can be used to find outliers. From the results obtained from the study, 20 outliers were found, where 12 matched the outliers found by other machine learning methods. The remaining 8 were reviewed by the radiologist, where 1 case was input error, 2 cases were measurement error (wrong slice was selected during markup). The remaining 5 cases were abnormal organ structure (sickle-shaped spleen, presence of additional lobules, other non-standard organ shapes in the form of a ball, triangle, etc.).

A general summary of outliers detected by machine learning methods is presented in Table 4.

**Table 4:** Number of potential outliers detected by machine learning methods.

Method	Diameter 1	Thickness 1	Diameter 2	Thickness 2	Diameter 3	Thickness 3
Isolation forest	6	1	1	0	2	1
DBSCAN	2	2	1	1	2	0
KNN	1	2	1	1	2	1
LOF	99 - Hard to interpret					
OSVM	5		6		7	
EllipticEnvelope	1	1	1	0	3	0
Autoencoder	20					

### 3.4 Revision of outliers, study interpretation

As mentioned in the Materials and Methods section, studies that were considered outliers were reviewed by an expert radiologist for correctness, input errors (e.g., incomplete numbers, incorrect field entries, unit errors), and organ abnormalities. Data are presented in Table 5.

**Table 5:** Distribution and interpretation of outliers determined by different methods.

Method	Input errors	Measurement errors	Abnormal values
z-score	3	3	3
Isolation forest	1	5	4
DBSCAN	3	3	2
KNN	4	3	1
OSVM	3	7	6
EllipticEnvelope	3	2	1
Autoencoder	4	9	8
Studies identified as outliers	4	15	13

OSVM-determined values differ from Table 4 due to some studies being marked as outliers twice by different radiologists.

Experts described abnormal spleen structure and size cases among abnormal values identified by mathematical statistics and machine learning methods: splenomegaly, abnormal spleen appearance, accessory spleen. The observations are presented in Supplementary Figure S2.

Thus, using a combination of machine learning, visualization, and mathematical statistics, we identified 24 patients with potential outliers, who were reviewed by radiologists. Some patients did have spleen abnormalities. The remaining diameter and thickness values were input errors.

### 3.5 Analysing AI performance

The percentage of hits in the spread before analysing outliers—were from 17.8 and 21.8% (in thickness and diameter respectively), after removing outliers—18.2 and 21.2%. We also analysed the performance of the algorithm solely with anomalous cases (no input errors and no measurement errors). It is noteworthy that the algorithm failed in measuring anomalous cases. Out of 13 cases that can be considered as anomalies of the organ structure or cases where the experts have established pathological condition of the organs, the AI fell within the range of measurements from radiologists in 7.7 and 23.1% of cases (in thickness and diameter respectively).

## 4 Discussion

The development and advancement of AI technologies present novel challenges for researchers and software developers. Oftentimes, these challenges necessitate the development of unconventional solutions. One of the main goals is to automate routine measurements and morphometry. Morphometry involves the application of AI models for automated measurement of dimensions, volumes, indices, and angles in diagnostic images. The training and validation of such models necessitate creation of reference datasets and development of robust evaluation methodologies. The curation of these datasets is associated with numerous complexities. This study focuses on a data analysis in morphometry: the identification of outliers in splenic size measurements obtained from abdominal CT scans.

The authors evaluated various outlier detection methods. The reviewed visualization techniques significantly simplify outlier identification and provide a comprehensive overview of the data distribution. The optimal approach involves a combination of calculation techniques and visual representation through graphs and histograms, complemented by tabular summaries of identified outliers.

The basic method involves constructing distribution histograms, enabling visual or boundary-based (e.g., 2*σ* or 3*σ* standard deviations) identification of outlying values. *Boxplots* are another commonly used data visualization technique. Scatter plots and heat maps are less common. The selection of a visualization technique depends on the specific task, data volume and type, and researcher preferences. The core principle of visual outlier detection is to define thresholds and analyse values outside them. These methods typically facilitate the identification of major errors, such as those associated with data entry or unit conversion.

More sophisticated methods, such as Z-scores, enable the identification of a greater number of anomalies and outliers, effectively detecting extreme values (e.g., in our study, 3 of 4 outlier errors were detected).

The Grubbs’ test enables analysis of extreme values and the derivation of statistically robust conclusions regarding outlier identification. However, this method presents several limitations in the context of medical data. Primarily, the Grubbs’ test is applicable only to normally distributed data. Medical research data often exhibit complex distributions, rendering this approach problematic. Furthermore, in medical contexts, values identified as outliers may possess clinical significance and necessitate careful review. Automatic exclusion of these values may result in the loss of crucial information. Considering these factors, for medical datasets, a thorough review of the maximum and minimum sample values informed by clinical context, may be more appropriate.

Rosner’s test, similar to the Z-score, is effective in identifying extreme values, but its application is also constrained by the assumption of normal data distribution.

Dixon’s, Chauvenet’s, and Romanovsky’s tests (Radkevich et al., 2006; Zalyazhnyh, 2022) were excluded from the primary analysis due to their efficacy being limited to small datasets. However, we assume that their application may be beneficial in other domains when addressing outlier detection challenges. In contrast to the other methods, which assessed outliers within the entire dataset (197 studies with diameter and thickness measurements from three radiologists), these criteria can be applied iteratively to each study and measured parameter. This approach facilitates identification of outliers in individual studies, which is particularly valuable in medical data characterized by case-specific nuances. These tests can be employed independently or in conjunction with other methods to pinpoint specific measurements that may be outliers. This approach was demonstrated in the studies (Promtep et al., 2022; Bendre and Kale, 1987) and proved effective in detecting anomalies in individual studies (Promtep et al., 2022; Bendre and Kale, 1987). However, most studies indicate that the Dixon test is less effective than other methods, such as Grubbs’ or Z-tests, for outlier detection across various distributions. It is most effective when applied to samples of 5–12 observations (Promtep et al., 2022). The presence of multiple simultaneous outliers can hinder the detection of individual outliers. Nevertheless, we applied the Chauvenet and Romanovsky tests to the data identified as “outliers” in this study, row by row. Using this approach, we did not detect any outliers. Therefore, within the context of this paper, these tests are not applicable, possibly due to the limited number of measurements per study.

Machine learning methods represent a complex suite of tools for outlier detection in medical data. Among the reviewed methods, only the Local Outlier Factor proved unsuitable for our dataset. Attempts to optimize the LOF algorithm resulted in an excessive number of outlier detections: 99 of 197 values were classified as anomalies. This may be attributed to the method’s high computational complexity, difficulties in hyperparameter selection, and reduced efficiency with small datasets, as the algorithm requires a sufficient number of neighbors for accurate density estimation. However, the literature suggests that 50–100 data points provide sufficient statistical significance (Popova, 2020).

OSVM was the most effective for outlier detection, identifying 16 anomalies, 9 of which were missed by other methods. Among these 16 outliers, 3 of 4 were data entry errors, and 7 of 12 were measurement errors. Additionally, the method identified 6 anomalies, including conditions such as splenomegaly and altered spleen morphology. However, interpreting its results can be challenging, as determining which measurements were classified as outliers and the underlying reasons requires in-depth expert analysis. Thus, OSVM demonstrated the highest sensitivity to organ structural anomalies among the other methods. This is because OSVM defines thresholds that separate “typical” data from outliers. It identifies a hyperplane in a multidimensional space that optimally separates normal observations from anomalies.

Isolation Forest ranked second in outlier detection, identifying 5 measurement errors and 4 abnormal values, but exhibited low sensitivity to extreme values (only one data entry error was detected).

The k-Nearest Neighbors (KNN) method demonstrated high sensitivity to data entry errors, detecting all 4 errors. However, it detected only one organ anomaly. The primary reason is that KNN is highly sensitive to inter-value distances. If the distance between data points is small, the algorithm may not classify a measurement as an outlier. This is because KNN relies on local data characteristics: it determines if an observation is an outlier based on the density of neighboring points. Consequently, in datasets with low variability or tight clustering, the method may miss significant anomalies, which limits its ability to detect outliers in complex or noisy data. Data entry errors are typically points distant enough from the main data distribution, which secures their identification.

DBSCAN demonstrated results comparable to KNN in outlier detection. With the settings described in the Materials and Methods section, this algorithm is effective in identifying extreme data anomalies. However, as the epsilon (*ε*) parameter decreases, the number of detected outliers increases significantly. Conversely, as the min_samples parameter increases, the number of outliers decreases. This is because DBSCAN classifies points as outliers if they lack enough neighbors within a specified radius.

The EllipticEnvelope method identified 6 outliers. With specific settings, it successfully detected 3 of 4 data entry errors, but the remaining anomalies were detected with reduced efficacy.

In total, we used the autoencoder to find 20 cases that were labelled as “outliers.” The advantage of this method was that it was able to find those abnormalities of the organ structure that were not detected by other machine learning methods. However, it missed some of the measurement errors. This method requires a large amount of resources (radiologists) to review values that are not detected by other methods. Additionally, it is worth noting that it can lead to over-analysis. However, research shows that with various ways to improve this method, it has great potential in medical applications (Zimmerer et al., 2022). Thus, it is best used in conjunction with other machine learning methods.

Approbation of the use of outlier search methods in testing the AI showed low values of hits in the range from three doctors, which is most likely due to two main factors. Firstly, peculiarities of the AI operation: during the analysis the expert noted incorrect segmentation of the organ (clipping of segmentation boundaries). Secondly, the peculiarities of measurement by doctors and AI. Radiologists perform measurements on two-dimensional images (slices), while the AI segments the organ. Post-processing of the AI results consisted in finding the maximum and minimum dimensions of the organ, which in practice does not always correspond to classical slice measurements. Therefore, counting the number of hits in the range before and after removal of outliers was not indicative in this case.

It is also noteworthy that the algorithm copes better with diameter measurements than with thickness. This is due to the fact that the diameter is measured from the maximum equidistant points of the anteroposterior dimension on the axial slice of the spleen, so it is not so difficult to find the mask with the maximum diameter. The thickness is measured from the gate of entry of the vascular bundle into the spleen, which does not always correspond to the minimum size and is much more difficult for this segmentation algorithm. A total of 13 analysed studies with abnormalities of the spleen structure showed that in this case the AI copes worse with the thickness measurement. The findings clearly demonstrate that the algorithms require additional training on abnormal data, which should potentially improve the performance of the AI.

It is crucial to recognize that in medical studies, the presence of outliers in measurements does not invariably necessitate data exclusion. This stems from the potential for such outliers to signify pathological changes in a particular patient. Therefore, measurements identified as outliers should be reviewed by a medical expert. The expert should decide whether to keep, replace, or exclude them from the dataset. This study demonstrates that variations in measurement methods are a primary source of outliers, highlighting the importance of developing precise labelling instructions for dataset curation. Such instruction must include measurement units, rounding parameters, measurement algorithms, and procedures for abnormalities, such as organ developmental anomalies, positional variations, and pathological changes. Including such studies in the dataset is essential, as it enhances representativeness and addresses anomalies and pathologies that demand urgent clinical attention, hence crucial for artificial intelligence development, testing and training.

### 4.1 Limitations

The methods evaluated were applied to two measurements of a single organ within one imaging modality (splenic diameter and thickness on abdominal CT scans). Results may vary under different research conditions. In addition, the normality of the distribution and the size of the sample under study should be taken into account when selecting the optimal methods for estimating emissions. When using machine learning methods, the hyperparameters are adjusted individually for each problem to be solved.

Future research should focus on developing automated medical image processing and analysis systems using integrated machine learning and statistical approaches. The results of this study (or the described methods) will be integrated in a data curation platform for visualization and automated outlier detection during study labelling (Vasilev et al., 2025). Such a tool will allow both an integrated approach to the creation of quality representative datasets and isolated use in analysing data for different scientific tasks.

## 5 Conclusion

This study investigated various methods for identifying outliers and anomalies in splenic linear dimension measurements obtained from computed tomography. The analysis revealed that both classical statistical and machine learning methods are effective in identifying data anomalies. OSVM and autoencoders were the most productive methods, identifying the highest number of outliers, though its interpretation necessitates significant expert effort.

Visual techniques, such as histograms and boxplots, proved useful in preliminary data analysis, enabling rapid identification of potential outliers. However, for a deeper understanding of outlier characteristics, algorithms such as Isolation Forest and DBSCAN, which provide detailed analyses and reveal hidden data patterns, are most useful. Statistical methods, such as Z-scores, can be effective in describing outliers but lack sensitivity to organs with anomalies. Notably, most established statistical outlier detection methods assume normally distributed datasets, which is often unrealistic in biomedical research.

It is crucial to recognize that presence of outliers does not invariably necessitate data exclusion. In medical research, they may reflect genuine pathological changes. Therefore, results classified as outliers or anomalies should undergo thorough expert analysis and review to inform subsequent data processing decisions.

This study underscores the importance of an integrated approach to data analysis in morphometric studies. It is evident that a universal approach or algorithm cannot be consistently applied to analyse such datasets. Only a combination of diverse outlier detection and visualization methods can enhance analysis quality and improve the reliability of conclusions, which is particularly crucial in medical practice.

The main task that is supposed to be solved with the help of the considered methods is the creation of datasets for testing and training of AI. First of all, it is to improve the quality of datasets (and as a consequence, AI) by handling measurement errors. In addition, these methods will identify anomalies and non-standard cases to add or remove them to the dataset (depending on the problem the AI is solving). Another important application area is population science research. The use of AI algorithms to process diagnostic images will produce large data sets that will be further analysed using the methods described. This will make it possible to identify and study non-standard cases, which in the future may lead to new scientific discoveries and improve the quality of medical care.

## Data Availability

The data analyzed in this study is subject to the following licenses/restrictions: the dataset is not publicly available because it is the property of the Moscow Healthcare Department. Requests to access these datasets should be directed to the corresponding author.

## Footnotes

^1^ https://mosmed.ai/ai/
