# Supplementary material for: Outliers and anomalies in training and testing datasets for AI-powered morphometry—evidence from CT scans of the spleen
Source: Front Artif Intell. 2025 Jul 15;8:1607348. doi: 10.3389/frai.2025.1607348 (PMC12303909; doi:10.3389/frai.2025.1607348)
Supplement: Supplementary file 1 [file Data_Sheet_1.pdf]

Supplementary Material

Supplementary Tables

Supplementary Table S1: **Basic descriptive statistics values differentiated by three radiologists**

**Descriptive statistics value**	**Thickness1***	**Thickness2**	**Thickness3**	**Diameter1**	**Diameter2**	**Diameter3**
Sample mean	48.2	46.0	47.2	97.5	100.6	101.1
Sample standard deviation	12.6	12.2	12.8	25.2	23.8	24.9
Median value	47.0	44.0	45.0	94.0	99.0	98.0
Maximum value	92.0	86.0	82.0	192.0	177.0	171.0
Minimum value	26.0	22.0	25.0	48.0	8.0	8.0
First quartile	38.0	37.0	37.0	79.0	85.0	84.0
Third quartile	56.0	53.0	55.0	108.0	113.0	113.0

* Index indicates the radiologist's number

Supplementary Figures

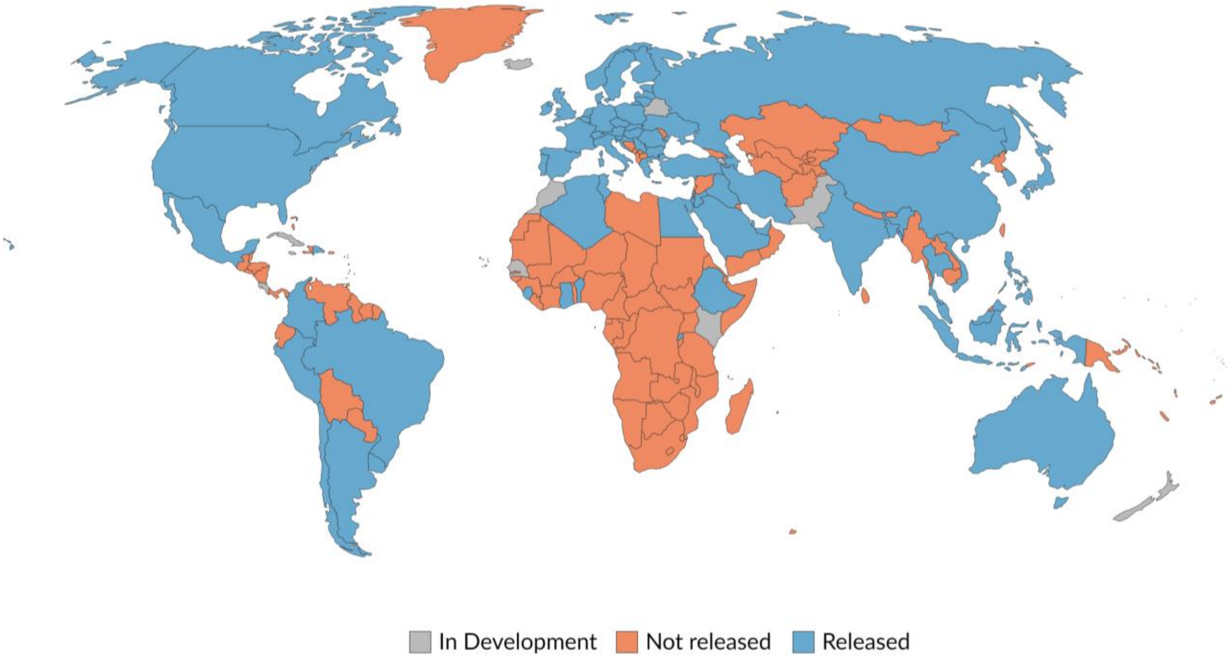

Supplementary Figure S1. Countries with national artificial intelligence strategies, 2023. An AI strategy is a policy document that communicates the objective of supporting the development of AI while also maximizing the benefits of AI for society. (https://ourworldindata.org/grapher/national-strategies-on-artificial-intelligence)

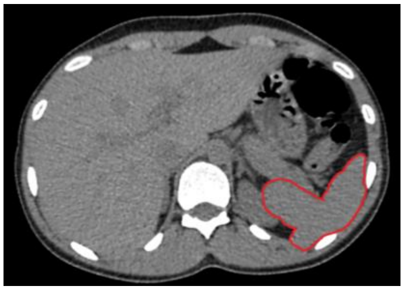

(A)

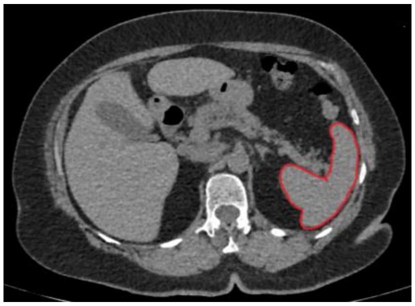

(B)

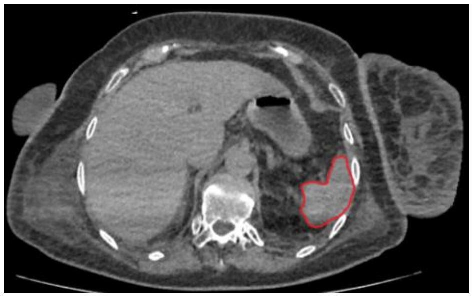

(C)

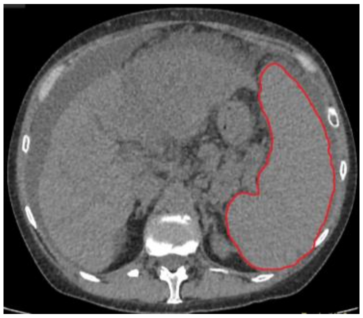

(D)

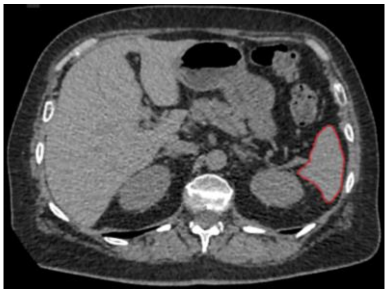

(E)

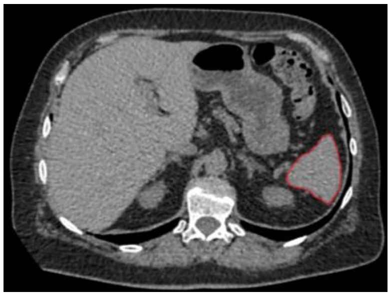

(F)

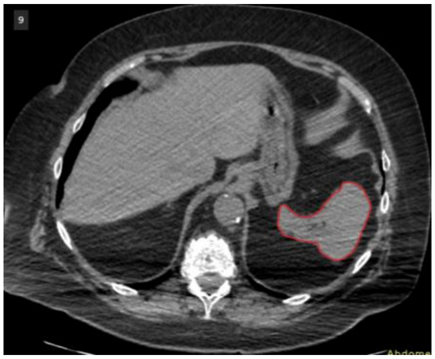

(G)

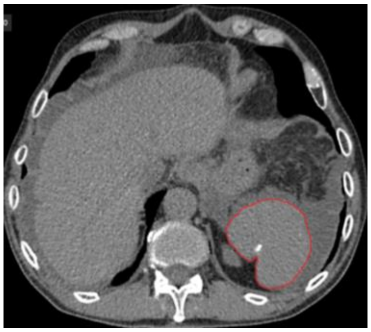

(H)

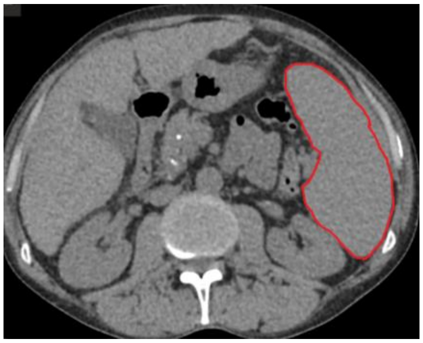

(I)

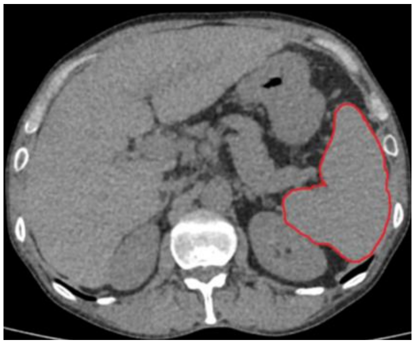

(J)

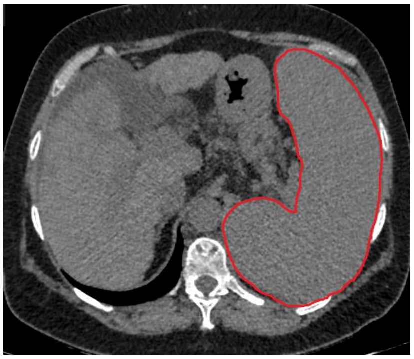

(K)

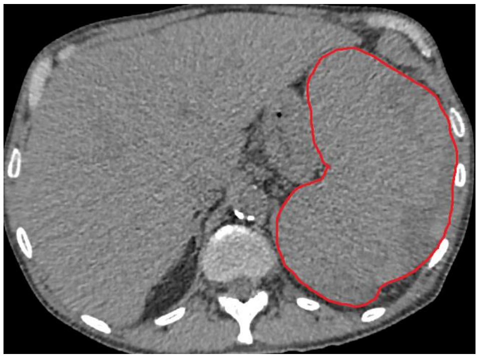

(L)

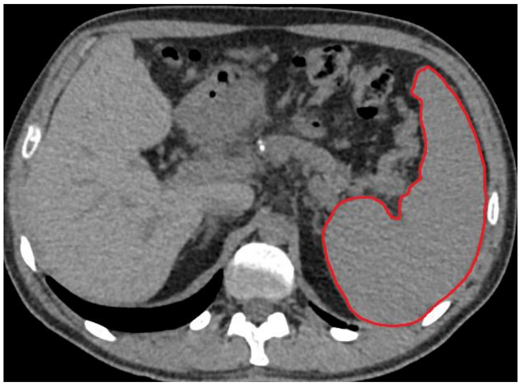

(M)

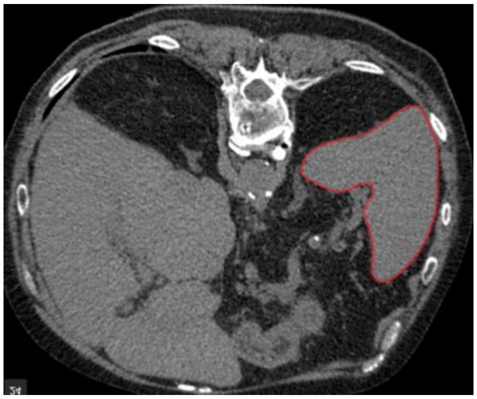

(N)

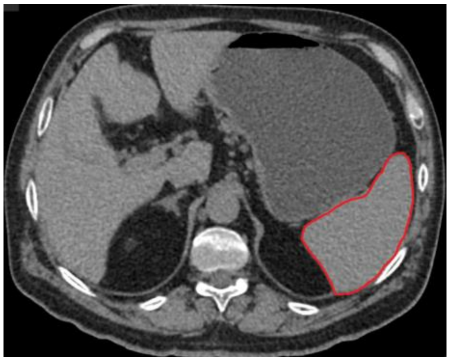

(O)

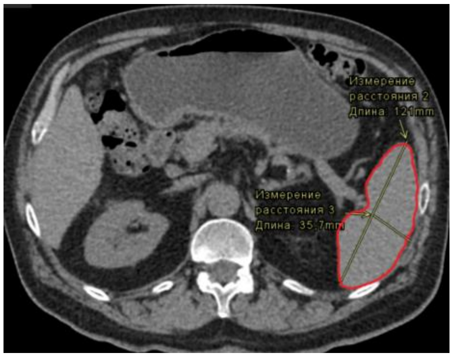

(P)

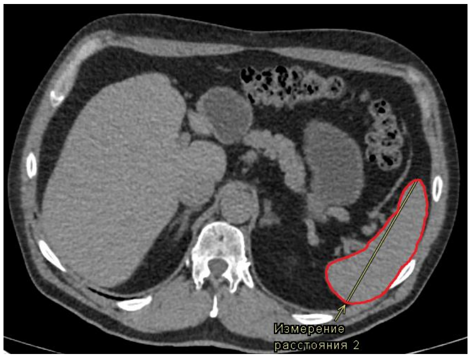

(Q)

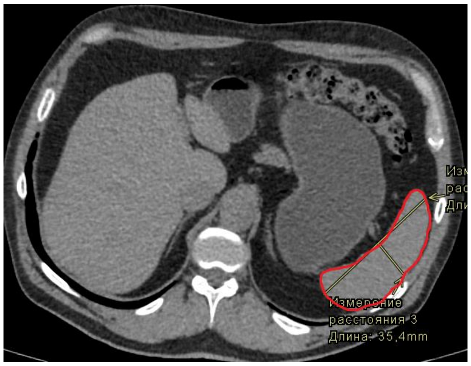

(R)

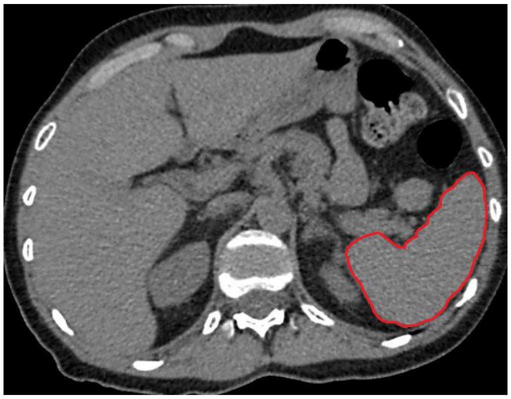

(S)

Supplementary Figure S2. Abnormal spleen appearance cases (highlighted in red) identified as outliers by different methods in 19 patients. **A** – developmental anomaly – accessory spleen; **B** – developmental anomaly – accessory spleen; **C** – small spleen, measurements are difficult due to anasarca; **D** – splenomegaly, ascites; **E** – not enlarged, abnormal triangular shape; **F** – not enlarged, abnormal triangular shape; **G** – abnormal appearance; **H** – abnormal round shape, ascites; **I** – splenomegaly, accessory spleen, measurements likely on different

sections; **J** – splenomegaly, accessory spleen, measurements likely on different sections; **K** – pronounced splenomegaly; **L** – splenomegaly, swollen spleen, ascites; **M** – enlarged, hook-shaped; **N** – abnormal spleen appearance; **O** – measurements likely on different sections, uneven spleen shape; **P** – measurements likely on different sections, uneven spleen shape; **Q** – moderate splenomegaly, measurements on different sections; **R** – moderate splenomegaly, measurements on different sections; **S** – hook-shaped.
